# Progress in Medicine

**Published:** 1898-05-21

**Authors:** 


### Progress in Medicine.

### DISEASES OF THE HEART AND CIRCULATION.

(*Continued from page* 114.)

**Iodides in the Treatment of Heart Disease.**—Vierordt[18] has recently reported the result of administering iodides, 15 to 25 grains a day, during long periods—many months—to patients suffering from arterio-sclerosis. Some of the patients were free from cardiac distress, but the majority suffered from angina pectoris. The effect was undoubted. In many cases the ability to work, which had been lost, was regained, and patients who had suffered from cardiac pain and faintness, and been unable to walk on a level floor, could now walk upstairs. Vierordt thinks that the morbid process on the blood vessels, which would otherwise always show a progressive character, is brought to a standstill, and that consequently the heightened blood pressure is gradually lowered, which would provide perfectly for the re-establishment of the circulatory function for a long time. The clinical phenomena do not indicate any retrogression of the vascular changes.

**Dropsy.**—Tyson,[19] in discussing the treatment of obstinate dropsy, after insisting on the importance of absolute rest in bed and limitation of the amount of fluids ingested, says the first thing is to obtain free movement of the bowels. This is best effected by saline purgatives. Rochelle salt in doses of half to one ounce in a minimum of water, say, four ounces, is very effectual. Epsom salt is, perhaps, more efficient but less palatable. This may be preceded by a largish dose of calomel the previous evening. After praising the diuretic power of digitalis, and of the old-fashioned pill composed of one grain each of squill, digitalis, and calomel, Tyson discusses the value of some newer drugs. He finds theobromine most effectual in the dose of 45 grains per diem. The drug is given dry on the tongue, and washed down by a draught of water. Theobromine acts better than diuretin, which is said to be composed of equal molecules of salicylate of soda and a compound of theobromine and soda. Next after theobromine comes sparteine sulphate, the active principle of broom; the dose should be from a quarter to two grains in the twenty-four hours. The author also finds baths given after the Nauheim plan a valuable auxiliary. At Nauheim the water is charged with carbonic acid, and contains certain saline constituents, mainly sodium chloride and calcium chloride. These baths can be imitated at home. For a bath containing 40 gallons 5 lb. of sodium chloride and 8 oz. of calcium chloride are required. To produce the effervescence 1 lb. of sodium bichromate should be first added and dissolved, and then 1½ lb. hydrochloric acid 25 per cent. The temperature of the bath should be between 80 deg. and 90 deg. The bath is a fairly satisfactory imitation except in regard to the carbonic acid, which is more rapidly dissipated in the artificial than in the natural bath; hence the patient should be promptly put into the bath after the hydrochloric acid has been added. The duration of the bath is eight minutes to start with; this is gradually increased to 15 minutes in succeeding baths. The immediate effects of the baths are a diminished pulse rate, intensified heart sounds, diminished breathing-rate, whilst the dilated heart is reduced in size. The effect is also to increase he action of the kidneys and skin. Massage is often a useful adjunct; a swollen limb, distended to almost stone-like hardness, is often rendered soft and flaccid by half-an-hour's massage, which seems to produce some absorption of the effused liquid.

**Prognosis in Heart Disease.**— Rejection of candidates for the army and public services sometimes takes place on insufficient grounds. Broadbent[20] points out some of the fallacies in making a diagnosis of heart disease in such cases. One of the reasons assigned for rejection is "irritable heart." Extreme nervous excitement is very apt to occur at such examinations; the pulse beats any number of times a minute, and perhaps not regularly, and the cardiac impulse is violent and extends over an unduly large area, perhaps lifting the sternum and giving rise to pulsation, to apparent pulsation, outside its right border. Whilst any proper appreciation of the sounds is impossible, and there may be murmurs at one or more of the orifices, the breathing is, at the same time, short and hurried. But such disturbance of action is not inconsistent with perfect soundness and efficiency. In such a case the personal and family antecedents are of extreme importance. If the boy is good at athletics there cannot be anything radically unsound about the heart. The condition is sometimes brought about by long hours at a crammer's for several months previously. Sometimes the simple expedient of letting the young fellow run up two flights of stairs will bring the heart to its senses.

A more common cause for rejection is the presence of a murmur perfectly innocent of significance. One of such murmurs is the spurious murmur not unfrequently heard in the apex region and to the left, which is produced by compression of the overlapping lung by the heart during systole. It simulates a soft systolic mitral murmur, but is easily recognised by the fact that it is heard only during inspiration, or whilst the chest is full. Sometimes this systolic exaggeration of the breath sounds is audible all round the left chest to the back; and, indeed, over a great part of the lung, but in this case there are usually pleural adhesions.

Again a murmur often considered to be of valvular origin may be sometimes heard in the pulmonary area, in the third left space, half an inch from the edge of the sternum. When at all loud it is usually heard over a great part of the right ventricle, and therefore in the tricuspid area, and may give rise to a suspicion of aortic obstruction. It is most capricious, often varying in intensity and character, sometimes only audible in the erect posture, more frequently only appearing when the patient is lying down. The important point about the diagnosis is that if the patient take a deep breath and hold it, the murmur disappears. It is probably due to the lung not coming sufficiently over the heart, so that the systolic bulging of the conus arteriosus of the right ventricle brings its anterior wall into contact with the chest wall, and sets up vibrations in the blood current within, thus producing the murmur. The murmur disappears as a rule when athletic exercises have expanded the chest.

Other murmurs may be heard over the tricuspid area which are harmless—one is a sort of systolic scratch which is readily recognised as exocardial; it probably corresponds to a white thickened patch often seen in the pericardium over the right ventricle. Again, there may be a systolic tricuspid murmur, but in these cases of primary tricuspid incompetence there is no evidence of any appreciable regurgitation as would be easily detected by jugular pulsation, enlargement of the liver, and distension of the right auricle. Not uncommonly it is obviously produced by distension of the stomach; it is probably due to the pressure on the yielding wall of the right ventricle where it rests on the diaphragm, which happens to take effect at a point corresponding to the origin of a papillary muscle. The adjustment of the valvular curtains is thus deranged, and a minute leakage permitted. Dilatation of the heart is not uncommon in boys who have engaged in violent forms of exertion, or who have been at cramming institutions and have taken all their exercise for the week at one time. The apex beat is outside and slightly below the normal situation and diffused, and the first sound of the heart is short. It is usually taken that such a condition is a permanent one, whereas it can be remedied by fresh air and exercise, and favourable hygienic influences aided by tonics. Dilatation of the heart as a disease, in the absence of valvular lesion or adherent pericardium, is unknown at this age except as a temporary consequence of diphtheria or typhoid fever, or acute rheumatism or other acute disease.

[18] Vierodt, Centralblatt für innere Medicin, 1897. [19] Tyson, Therapeut. Gazette, Jan. 15, 1898. [20] Broadbent, Clin. Jour., Oct. 27, 1897.

### DISEASE OF RESPIRATORY ORGANS.

(*Continued from page* 115.)

**Open-air Treatment of Phthisis.**—Von Ziemssen,[16] finding the R. tuberculin useless, fell back on open air. In high altitudes the air is dry and pure, the sun's rays warm, and there is an absence of fog, but he does not think the increase of red corpuscles observed is of therapeutic importance, and he advocates the formation of sanitoria at any level where open-air treatment can be given, as this is of more importance than the altitude. Special interest has been excited in this subject by the papers of Burton Fanning and others on the success of open air sanitoria in this country. The *Lancet*[17] remarks that the discovery that such treatment can be carried out with success in this country and that patients can remain in their native land should produce very great results. Fanning[18] has experimented with a small home at Cromer, where the patients go out of doors to a verandah screened from the wind and remain out all day. Plenty of rugs, hot bottles, and padded foot bags are provided. Absolute rest is enforced for those who manifest any weakness ot the pulse, which he regards as an essential in the treatment. No routine drugs are given, but a nutritious diet is ordered, and care is used to get rid of the expectoration. The nights are passed in a large room with open windows. Out of 24 cases he reports two cured, four relatively cured, 12 improved, and six only temporarily improved. The open-air treatment, he adds, has given him better results than any other, and it is followed by a decline of fever, though it should not be attempted where the morning temperatures are over 99·5 until this has fallen. The cases do better in cold weather than in warm, appetite rapidly improves, sweating disappears, though cough may at first increase. F. Pott has also organised a house at Bournemouth for well-to-do patients, who live practically out of doors in verandahs and shelters on the lawns. As to the provision of general homes and sanitoria for the phthisical, Lindsay[19] remarks that the deaths from phthisis in the United Kingdom amount to 60,000 per annum, and there are only sanitoria for about 1 in 200 of those suffering from the disease among the poorer classes. On the other hand, if we ask whether such hospitals would be of any use, we find in the Falkenstein sanitorium as many as 30 per cent. of absolute and relative cures are claimed; Weber notes 24 per cent., Allbutt 22, and Spengler 21 per cent. at high altitudes, and not only are such homes important from their direct results, but they educate the patients in proper care of themselves, and lessen the risk of infection.

**Infection.**—Flügge[20] thinks that infection by dust from dry sputum is not proved, and is, indeed, improbable, but the finely divided fluid sputum produced by coughing is a real danger, though easily avoidable with care, and not a frequent cause of infection. G. F. Gardiner[21] discusses the actual causes of the exemption from phthisis which is found at high altitudes, which he has made the subject of elaborate investigations. The exemption is not quite absolute; a very few cases of the disease do arise in such places as Colorado, and are generally rapid and fatal, but practically dwellers at such an altitude are immune. Now he finds that non-pathogenic germs exist in numbers in the dust of Colorado City, though far out on the prairie there are absolutely none. Germs, too, grow well in cultivation media as at low altitudes; sputa, when inoculated, even if dried in the sunlight, produces the disease, but dust from rooms failed to do, and, indeed, septic results rarely follow. Thus he thinks the tubercular germs are rapidly killed in the dry, sunny climate, where the air is so thin. The lungs are more vigorously used from the rarity of the air, the specific gravity, red corpuscles, and hæmoglobin of the blood are, as is well known, markedly increased, giving greater germicidal power and more rapid tissue change. The climate acts as a tonic to the nervous system, and the dryness of the air and soil are also important factors in causing the immunity, and, lastly, the practical absence of tubercular disease in the cattle reduces the chance of infection from milk. On the whole, then, he shows that tubercular infection, in spite of a large population of imported invalids, is rapidly destroyed by the climate, and the individual is better able to withstand it.

A. Ransome,[22] in writing of consumption as a filth disease, shows that it is spread by the "air sewage," or the matters excreted by patients into the air; and secondly, by the growth of the germs, which takes place outside the body in dark, damp, unventilated houses. It is not indeed due to filth alone, but to that which contains and affords a growing place for the specific germ. Still, he shows that most nitrogenous substances, with the addition of glycerine, are good cultivation media, and moisture from the breath, or from cellar air, or from the air of crowded rooms, also forms a good medium, even at the ordinary external temperature. Thus theatres, cellars, churches, railway carriages, as well as dark, damp, ill-ventilated cottages, are veritable gardens for the growth of the tubercle bacillus, infecting those whose houses are in perfect sanitary condition as well as those whose habitations are themselves infected.

Does phthisis ever follow in consequence of an injury? O. Schrader[23] mentions an instance where a man fell on his back with great force, and was admitted to a hospital next day with pneumonia. A remittent fever followed, and tubercle bacilli were soon found in the sputum. There may have been latent tuberculosis, or infection may have taken place after the injury, but he shows strong reason to think that the disease was actually produced by the injury. J. H. Larkin[24] reports finding caseous degeneration and virulent bacilli in lymph nodes from the lungs of a patient who died of chronic bronchitis and emphysema, without any other signs of tubercle in the body. He points out that the nodes thus not only take up noxious matters, but also bacilli, and that in some cases the body may be infected from them when a favourable chance occurs. It has even been asserted that phthisis when shown by physical signs is really in the second stage, the poison having entered first through the alimentary canal and mesenteric glands.

It is undoubtedly of the highest importance to recognise lung disease in the very earliest stage. If there be a suspicion of dulness, a small area of rough breathing, or prolonged expiration, we should certainly watch the patient carefully, and C. P. Ambler[25] advises that in such cases the temperature be taken every two hours. If a daily rise of one or one and a half degrees is found, the evidence is strong for an early stage of tuberculosis in his experience. Douglas Powell[26] discusses a case of ascites in an aged man, where the ordinary causes could be excluded, but who showed some signs of clear phthisis in one apex. The diagnosis was chronic tubercular peritonitis, for, in addition to the other evidence, there was some abdominal tenderness, while percussion showed that the intestines were bound down in the flanks, and pushed upwards in the liver region, so that the proper dulness of the latter organ almost disappeared. Cancer, cirrhosis, and other affections could be excluded, and with rest in bed, good food, and creosote the patient quickly lost nearly all symptoms of his disease.

The microbes associated with the tubercle bacillus in diabetic phthisis seem, according to Ehret,[27] to differ from those in ordinary cases. Thus, besides strepto and staphylococci and tetragoni, he finds a pseudo-diphtheria bacillus, which flourished on a saccharine medium, forming punctate colonies in 24 hours. Possibly this is the cause of the rapid course of phthisis in diabetes.

[16] B. Med. Jour., Feb. [17] Lancet, April 2. [18] Lancet, March 5. [19] Lancet, Dec. 4. [20] B. Med. Jour., Jan. 22. [21] Amer. Jour., Med. Sc., Feb. [22] Lancet, Jan. 1. [23] Amer. Jour. Med. Sc., Feb. [24] Med. Rec., Dec. 18. [25] N. Y. Med. Jour., Feb. 12. [26] Middlesex Hosp. J., Dec. [27] B. Med. Jour., March 19.

